# Advances in Perioperative Management

**DOI:** 10.1155/2014/246267

**Published:** 2014-06-16

**Authors:** Alaa Abd-Elsayed, Elizabeth Frost, Ehab Farag

**Affiliations:** ^1^University of Wisconsin School of Medicine and Public Health, Madison, WI 53792-3272, USA; ^2^Icahn School of Medicine at Mount Sinai, New York, NY 10029, USA; ^3^Cleveland Clinic, Cleveland, OH 44195, USA

There has been great progress in perioperative management modalities and technologies aimed at improving patient care and safety. This issue includes very important articles in this field. An article by R. Gharabawy et al. discusses the efficacy of ambulatory nerve catheters. The study was done on a large number of patients and showed the safety and feasibility of placing catheters on an outpatient basis. The use of ambulatory nerve catheters aims at reducing admission costs and improving pain control and thus patient satisfaction. Another paper by L.-E. Kang et al. discusses the outcomes related to the use of different cuff pressures of supraglottic airways during abdominal laparoscopic surgeries. This article confirms the safety of using the laryngeal mask airway (LMA) in these surgeries. An article by I. Son et al. presents a double blind randomized controlled trial of the effect of sufentanil administration on remifentanil-based anesthesia during laparoscopic gynecologic surgery. In the article by T. Gaszynska et al. the efficacy of endotracheal intubation using Levitan FPS optical stylet versus LaryFlex video laryngoscope in morbidly obese patients is compared. There have been a lot of advances in airway management in anesthesia, which is not surprising, as intubation/airway management can be life-threatening. It is very important to have multiple modalities available to avoid complications during intubation. Another article by E. Gaszynska et al. reviews a very important topic: satisfaction among anesthesiologists, a critical factor in ensuring better patient outcome. Aside from technology and equipment, we must pay attention to the human element. The article by N. Mehta et al.,* “A review of intraoperative goal-directed therapy using arterial waveform analysis for assessment of cardiac output,”* considers also noninvasive monitoring. Such technology is rapidly becoming the standard of care for fluid replacement. The article by S.-H. Kim et al. is a double blind randomized controlled trial that shows that total intravenous anesthesia with high-dose remifentanil does not aggravate postoperative nausea and vomiting and pain, when compared with low doses.

*Alaa Abd-Elsayed* *Alaa Abd-Elsayed* *Elizabeth Frost* *Elizabeth Frost* *Ehab Farag* *Ehab Farag*

